# Indents

**Published:** 1910-10-15

**Authors:** 


### INDENTS.

**☞Brief Articles of Technical or Scientific Value will receive notice and comment. Contributions invited.**

*Dentistry Legalized as a Specialty of Medicine.* Virginia has just enacted a new dental law that is a radical departure from those in other states as it legalizes dentistry as a specialty of medicine.

Sec. 2 of the bill reads as follows:

"From and after January first, nineteen hundred and fourteen, Anno Domini, the practice of this specialty (dentistry) in this state shall be a branch or specialty of medicine and surgery, and no person, after this act goes into effect, shall be given the examination or a certificate required by section four of this act unless he shall first show to the satisfaction of the examining board provided herein that he has passed the examination provided by law for applicants to practice medicine or surgery and has received from the Virginia state board of medical examiners the certificate thereof as required by law to be given by them to such applicants."

Other features of the law are, that a board of dental examiners, consisting of three practitioners of dentistry of acknowledged ability in the profession, shall be appointed by the Governor upon recommendation of the Virginia State Dental Society. The member of the board to hold office for three years (after the first appointmemt), one member going out of office each year and his successor to be appointed by the Governor from two persons nominated by the Virginia State Dental Society.

All licensed dentists must register with the board each year.

The examination fee will be two dollars for each examination and the registration fee will be one dollar for each person registering.

Physicians or any other persons are permitted to extract teeth for anyone suffering from toothache and bona fide students in regular attendance at any dental college in the state may practice dentistry, under direct supervision of one of its teachers, in the regular infirmary of such college.

The law is on the statute books. Now, can we live up to it? Can we teach dentistry as a branch of medicine for dentists, along with other medical studies (letting, for the time being the medical student leave out dentistry) in four years, taking dental branches in vacation or post course, or by adding another year?

It will be a hard course and only the demand for dentists would make it seem desirable to a student. Medicine plus dentistry, is what it means. If the student is allowed to specialize, as he should be, it will not be so hard. Just this. The way must be found. Our best men must get together and formulate some plan of study.—Dr. F. W. Stiff in *Summary*.

*Clay Pipes and Gold Inlays.*

1st. Procure from a dealer in smoking goods, a dozen common clay pipes, that retail for a penny each. You can obtain larger ones that are two or more inches across the bowl; these are used in large bridge castings.

2nd. Obtain some soft copper wire, about 14 gauge, wind one end five or six times about a pipe stem, near the middle, bend the remainder down at right angles for about four inches and fasten to a block of wood four inches square by one in thickness. You now have a device which holds the pipe bowl up and level.

3rd. Obtain a good bicycle foot-pump, one in which the valve at end of plunger can be removed and reversed or inverted, thereby making it a suction pump.

4th. Get three or four feet of small heavy tubing that will not easily collapse when suction is applied. Fasten one end of tubing to the pump in place of the usual short tube which accompanies the pump, wire or tie the tubing in place.

Slip the other end of rubber tube on the end of pipe stem and when you pull up on the pump plunger or handle you should get a strong suction, causing the pipe to stick to the palm of your hand, when held over the bowl.

I would advise you to fasten the pump underneath your workbench by two strips of tin or leather bent over the barrel and tacked underneath bench, have the handle in a convenient position to pull out when you are ready to cast. The rubber tubing should be brought up behind the bench and held with a staple where it is always handy to slip over a pipe stem. Mounted this way your Casting Machine takes up no valuable space, is always ready for action. Your outfit is now complete and there is no other that can give you more perfect results than this, when once you have learned how to use it.

The idea is to make a pipe serve as an investment ring or cup, also act as a casting machine, it being clay, stands the heat of drying out and melting of gold.

To invest the wax in pipe bowl, first place a little asbestos wool over the hole to prevent investment filling the stem of pipe, then place the pipe in water, this prevents it from drawing the water out of the investment. Mix investment and paint carefully over wax, fill the pipe bowl with investing material and place wax and sprue former into pipe, let it harden a few minutes when the sprue wire may be heated and removed. Cut a slight concavity about sprue to hold the melted gold, dry out slowly till all moisture and wax have burned out, place pipe in the wire holder, slip end of rubber tube over pipe-stem, see that pipe is level and handle of pump pushed in. Place a nugget of gold into concavity of investment, melt to a white heat, pull slowly out on pump handle and hold till gold cools, a cold instrument applied to excess of gold will hasten cooling. Your cast is now made, finish in usual way.

Two or more inlays may be cast at the same time, a crown or two bridge dummies may be cast in an ordinary pipe, larger pipes for larger bridges.

For very rapid inlay work, the pipe bowl should be cut off with saw, about a third of its length, thus requiring less investment which can be dried out in twelve minutes ready to cast into.

I have used about all the machines made, and this is the best, simplest, smallest, most reliable casting device I have ever used regardless of price. Try it.—C. S. Starkweather, in May *Summary*.

*A New Principle in Difficult Bridgework.* To overcome unparallel abutments for bridge work, making a sanitary bridge, and at the same time not destroying the teeth in order to gain parallel walls. Where two or more dummies are required, as for lower bicuspids and molars, make caps for abutments, and metal dummies with a hinge joint which will permit the flexion of the bridge upward while cementing to place, and permit its placement in proper occlusion.

The bridge is made sanitary, hinge is hermetically sealed by placing a layer of fiber gold in the hinge just before the bridge is set, afterward burnishing the same.—Dr. W. H. Vallender in *Dental Summary*, March 1909.

*School Children's Teeth in New South Wales.* (1) During 1908–9, dental records of 3996 children in country districts were received from the Dental Association of New South Wales.

(2) The teeth of girls in the country districts are more defective than those of boys. This was found to be the case also in Sydney.

(3) Defects are much more common among teeth of the first set than among permanent teeth.

(4) The first teeth among country children are slightly more defective than those of metropolitan children, but country children have much better second teeth than have city children.

(5) In the use of the tooth brush, boys are more negligent than girls, and the cleanest teeth and mouths are found among country girls.

(6) Of 3574 country children examined, 23 per cent. were found to be of blonde type of appearance, 20 per cent. dark or brunette, and 57 per cent. of medium type. In Germany the corresponding percentages are 32, 14 and 54.

(7) From dental statistics received so far, children on the Western Plains (Narrandera, Hay, and Bourke) are shown to have better teeth than the more robust children on the Tablelands.

(8) The medical inspectors' reports all points to the fact that the teeth of school children are much neglected.—*Cosmos* for July, 1910.

*Cleaning Pyorrheal Pockets.* First of all the tartar is removed from the roots without injuring the sockets of the teeth. Perfect success depends not so much on the set of instruments used as on the operator's skill. The process is a lengthy and difficult one; the calcarous deposits at the necks of the teeth are removed with a very fine fissure bur, and the teeth polished afterward with a minute cone-shaped bur. Then perhydrol is forced into the pockets. The method of applying it consists in dressing the end of a small horse-hair pencil with a soft sticky-wax, in order to keep the hair together and to render the brush stiff. The brush is dipped into the perhydrol, and by a rotary movement introduced into the pockets. It is washed and wiped every time before being dipped again into the perhydrol. After the first treatment the pockets will fill with white pus, which is removed by a water spray. The pus grows less after every application, and the gingiva are given a strenuous massage with the finger-tips, starting from the apex of the root toward the crown of the tooth. This massage is repeated daily, and the patient supplied with a brush and diluted perhydrol in a well-corked bottle. Energetic use of a tooth-brush without powder is advised, or instead of the dentifrice table salt may be used on the gums and teeth. If applied at an early stage this treatment permits of a favorable prognosis, and a great many teeth can be saved.—Jens Boberg, *Tandlaegebladet.* (Perhydrol is a highly concentrated hydrogen dioxid preparation (30% absolute H2O2) made by Merck.)—Ed., *Dental Cosmos.*

*Dental Prophylaxis Aids.* For polishing the natural teeth after cleansing, Dr. Goodman A. Miller, of Chicago, recommends chemically pure oxid of tin, slightly moistened with wintergreen or any pleasant flavored liquid. It may be used with the hand port-polisher, or on moosehide, or other polishers in the dental engine. It quickly imparts a high polish. In the port-polisher he uses a good grade of shoe-pegs, finding them inexpensive and convenient.—*Dental Summary*, December 1909, page 969.

*Dentists Needed.* Dr. Harry Campbell, presiding at the annual meeting of the National Food Reform Association at Caxton Hall, said a result of improper dietary was the condition of the teeth, which were, he declared, a national disgrace. A certain philanthropist had recently given £200,000 with the object of providing dentists to look after the teeth of the people of this country. He ventured to say it would cost more like £300,000,000 to attend adequately to the teeth of the people of this country. It would mean one dentist to every 1,000 inhabitants to look after the teeth of the people in their present condition, and would accordingly represent 1,000 dentists to every million persons, or 40,000 to the entire population. It would therefore ,take an investment of £300,000,000 to provide a revenue of £300 a year for each of those dentists, which, he believed, would be the minimum average salary the dentists would accept. The dental trouble was really a question of diet. —*Dental Record.*

*The Dentist who is "Past Fifty."* In order to advise the man "past fifty," one would have to know something about his mode of practice, and how he keeps his office. Do things about him look neat and clean? Does he have attractive things in his reception room? Is he surrounded by everything new and up-to-date and can he do the work in a proper manner? Is the technic of gold and porcelain inlay work too much for him? Can he make teeth that look as they ought to? Can he fill a tooth properly with gold, treat and save diseased teeth, including our old friend pyorrhea, and can he as a last resort extract without a trembling hand any tooth or root?

If he runs his office in a first-class manner with his experience back of his ability, and is genial with people, has a reputation of being "on the square" and ethical—if the people don't patronize him, and go trailing after newly fledged tyros, he can't help but have any good man's sympathy and a bracing word, for he is just in his prime, but must not stand still. This is written by a man past 65.—Dr. C. B. Plattenburg, in *Dental Brief.*

*Unlawful Method of Collection.* In a trial at Paterson, New Jersey, of a suit brought by Dr. William Schanyerson against Mrs. Samuel Champagner, to recover $50 for dental work, it developed that the dentist after inserting upper and lower bridges, removed them as the woman sat in the chair, because she was unable at once to make payment in full. She said she had paid $50 when she ordered the teeth. The two sets of teeth had been cemented in position, when the dentist asked for the final payment. "You can have it next week," the woman told him. "That won't do," he declared, as he forced the teeth from their setting, according to the woman's testimony before Judge Lewis. The dentist admitted that he removed the teeth, and Judge Lewis decided against him, holding that his claim did not hold good in law until after he had completed his work.

*Prolonged Nitrous Oxide Anesthesia* A case which occured recently at the Royal Dental Hospital of London is of considerable interest, in that in this instance a patient was kept under gas for close upon forty minutes, and was able to leave the hospital within half an hour of the termination of the dental operation to which he had been subjected. If a method could be advised by which gas could be used conveniently and cheaply for long operations, it would doubtless be a great boon to surgeons.

*A Duty of the Dental Profession.* The time has now come when dental hospitals should be established either independently or as departments of existing hospitals, and accessible at all hours to those suffering from dental lesions. The dental hospital should be a practical educator, conducted in such a way as to educate the people to appreciate good dentistry and its value. Free hospital clinics only educate the people to look for the best that man can do for nothing. Wholesale charity pauperizes the giver as well as the receiver. All these things should be considered in the management of the dental hospital, and each and every patient should be made to feel the real value of what they receive, if they pay for it or not; and all should be made to pay in proportion to their ability to pay.—Alice M. Steeves, D. D. S., Boston, Mass., *Items of Interest*, December, 1909.

*Hot-Air Therapy.* The merits of hot-air therapy in dentistry, as applied for desiccation, antisepsis, hyperemia, hemostasia, and anesthesia, are reviewed with special reference to the degrees of heat recommended for various purposes by noted recent authors on this subject. The authors discuss the merits of hot-air treatment as applied by themselves in the following four ways:

First: Cauterization at low temperature (50° C.) with slight pressure, for the desiccation of vital teeth.

Second: Cauterization at high temperature (from 110°

to 120° C.), applied under high pressure and prolonged insufflation, lasting from seven to ten minutes, in teeth with dead pulps, for sterilization of the dentin and hyperemia of the surrounding tissue.

Third: Cauterization at high temperature ( bout 120° C.), under high pressure, for the production of hyperemia by insufflation upon the gingivae in cases of pyorrhea, stomatitis, and fistulas.

Fourth Cauterization at very high temperature in cases of hemorrhage—*Cosmos.*

*Sulfurous Acid $H^2SO^3$) in Dentistry.* Sulfurous acid, which has been used for various purposes in general medicine, has been tried by the author in the treatment of pyorrhea alveolaris with good results. The mode of application is as follows: After the mechanical removal of the tartar, the pat ent's teeth are painted with H2SO3, and the following mouth wash is prescribed for daily use: Sulfurous acid, 60 per cent., from 10 to 20 drops to be diluted in a small glass of water. In this dilution the acid secms to have no ill effect upon the structure of the teeth The author merely wishes to draw the attention of the profession to this remedy, claiming for it special merits in the disinfection of prosthetic pieces. Any therapeutic agent applied for this purpose must be such as to exert no deleterious influence on the teeth and the mucosa. Moreover, it should be of reasonable cost in order to lend itself for daily use by all classes of patients.

From twenty to thirty drops of the acid are mixed with water enough to cover the prosthetic piece, which is left in this solution over night and carefully cleaned with water in the morning. If kept in a covered vessel this solution may be used for a week. The effect of H2SO3 upon the rubber is most favorable, as it imparts to it a natural-looking, pale tint. Moreover the evil odor of the prosthetic piece is not merely disguised as in the case of mouth washes, but is really destroyed entirely. No trace of the tartar that is so frequently found on rubber plates is left, and the main objection that many patients have to a prosthetic piece, namely, that of the difficulty of keeping it aseptic, is obviated.

On account of the chemical properties of H2SO3 it is advisable to prescribe only very small quantities of it at one time.—J. K. Pedley, in *Cosmos.*

*Alcohol and Dental Caries.* Dr. Floxas, of Constantinople, has examined the teeth of 729 Mohammedan workmen, 198 of whom were no longer total abstainers, with the following results:

*Percentage of Dental caries.*

| Ages. | Abstainers. | Non-abstainers. |
|---|---|---|
| 15 to 20 | 0 .5 | 1 .9 |
| 21 " 25 | 1 .0 | 3 .7 |
| 26 " 30 | 2 .0 | 4 .8 |
| 31 " 35 | 2 .7 | 7 .2 |
| 36 " 40 | 4 .8 | 10 .4 |
| 41 " 45 | 5 .4 | 11 .6 |
| 46 " 50 | 4 .4 | 17 .2 |

These differences are so striking, and the number of cases examined is so great, that these results cannot be simply accidental. Dental caries, besides many other symptoms of degeneration, is the result of chronic alcohol poisoning, alcohol of course not being the only enemy of sound teeth, but a specially obstinate one. Abstinence from alcohol, therefore, is not only a great and economic preventive of dental caries, but will have especially beneficial effects on the third and fourth generations.—*Deutsche Zahnaerztliche Wochenschrift.*

*Methods of Hardening a Plaster Cast.* Apply a hot saturated solution of borax or alum with a brush, giving the cast two or three coats to insure a permanent hardening of the outer surface; then apply two coats of a hot saturated solution of baruim chlorid, followed by two coats of soapy water, washing off the surplus soap with clear water. This process produces a hard surface that is insoluble in water, and which will preserve the cast indefinately. If the plaster of Paris is mixed with gum arabic before the cast is made a coating will not be necessary, as the cast will be hard and durable and have a very smooth surface.—John Schembs, Jr., in *Cosmos.*

*Treatment with Vaccine for Loose Teeth.* One of the recent interesting developments of opsonic methods is the application of them to pyorrhea alveolaris —loosening of the teeth—a subject that is discussed by Dr. Leon S. Medalia, in a recent issue of the *Boston Medical and Surgical Journal.* The work is on autogenous principles, for since the causes underlying the disease are still very obscure, it has been possible to make from the pus of the gums of the patient a bacterial vaccine that can effect a cure. The work was begun while Dr. Medalia was connected with the Tufts Medical School, and has since been carried on in the clinics of the Mt. Sinai Hospital, and in private practice among his own patients and those of other practitioners following the same lines of treatment. The number of cases is now above one hundred and fifty; small, to be sure, for the certain establishment of so important a principle, but affording in many instances more than a year in which no recurrence of the difficulty has appeared. The initial case was pyorrhea of five years' standing, advanced, and the patient had lost three teeth, two were slightly loose and a gread deal of pus was present on the gums. Without other treatment than an autogenous vaccine produced from the pus, improvement began at the end of one week and cure was effected in six weeks, with no recurrence. All of the other cases have shown marked improvement. The conclusions advanced by Dr. Medalia are first, the principle already accepted, that pyorrhea is an infectious disease, that the opsonic finding is peculiar and indicative, and that cases that are sufficiently far advanced to produce pus yield in good proportion to vaccines, preferably autogenous. In this connection the writer points out that certain tired feelings and some of the articular rheumatisms may come into a class similar to the dental troubles and these in the patients suffering with pyorrhea have yielded to a degree at least to the inoculatory treatment.—*Boston Transcript*, April 6, 1910, —*Brief*, August, 1910.

*Dictated But Not Read.* What a silly postscript, "Dictated but not read." The sender would have you believe that he is a very busy man, with a correspondence so vast that he is for want of time unable to read the note he dictates and signs. He had far better keep that to himself. Signing without reading now and again proves a costly negligence; and a note the writer has not taken the trouble to read, and tells you so, deserves a rapid transit to the waste basket.—*Brief.*

*Mastication, the Problem of.* The most important matter in healthy life-sustaining mastication, and the most pressing one to many, is something life-sustaining to masticate. The teeth, important as they are, are secondary. One can "gum it" on a pinch. Indeed, one distinguished dentist contends that store teeth are better for those somewhat advanced in age, they are easier to keep clean, and being less effective, interdict tough beefsteak and the like, which old folks, he contends, have no business to tackle.—Trueman in *Brief.*

*How to Fix Fees.* Dr. John DeHaven White, in a business lecture to dental students, gave this advice on the fee question: Your first and most pressing want when you enter actual practice, is patience you want to get to work and keep from rusting. Never mind the fee, get patients, even if you have to pay them to come to you. When the tide turns and comes your way then it is your turn. Get busy for what you can get until patients have become so attached to you and your work so firmly that they think no one in this wide world can do their work but you; then charge as much as your conscience permits, but be sure you have a firm grip before running up the charges.—Trueman, in *Brief*.

*Sciatica and Bridgework.* Arthur S. Underwood, M.R.C.S., L.D.S., reports in the *British Journal of Dental Science* for March 1st, having been called upon by the patient's physician to examine the mouth of a man suffering from sciatica. The case had not responded to treatment, and the physician was impressed that some undiscovered trouble was the cause, and wished to know if any complicating mouth conditions existed. He found an extensive fixed bridge in position, constructed and inserted by a Hungarian dentist some twelve months before. This to all appearances was in good condition. The patient reported that although its construction had been a very painful operation owing to the necessity of grinding the surfaces of live molars when fitting the supporting caps, since it had been installed it had been quite comfortable, and was used freely, and with satisfaction in mastication. He was, however, conscious at all times of an unpleasant taste, which he referred to general dyspepsia. Mr. Underwood reported to the patient's physician, that while the bridge might afford a nidus for bacterial activity, as it was painless and serviceable he hesitated to remove it. After consultation it was decided to remove it. This was done in June, and a shocking septic condition was revealed of which the mouth before the removal of the bridge gave no suggestion. The mouth was thoroughly cleaned, and the missing teeth replaced by a suction plate. The sciatica and all the general systemic conditions immediately disappeared, and the patient's physician was able to give him a clean bill of health. The point in this case was, that although the bridge was well designed and well executed, and for a year had done good service, enabling the man to masticate without discomfort, and beyond a slight odor gave no signs of anything being amiss, there existed underneath this bridge a germ factory of great activity that was slowly but surely poisoning the whole system.

